# Higher carbohydrate intake in relation to non-alcoholic fatty liver disease in patients with type 2 diabetes

**DOI:** 10.3389/fnut.2022.996004

**Published:** 2022-12-08

**Authors:** Nora A. Alfadda, Ghadeer S. Aljuraiban, Hadeel M. Awwad, Mohammad S. Khaleel, Abdulrahman M. Almaghamsi, Suphia M. Sherbeeni, Adel N. Alqutub, Abdullah S. Aldosary, Assim A. Alfadda

**Affiliations:** ^1^Department of Community Health Sciences, College of Applied Medical Sciences, King Saud University, Riyadh, Saudi Arabia; ^2^Obesity Research Center, College of Medicine, King Saud University, Riyadh, Saudi Arabia; ^3^Obesity, Endocrine and Metabolism Center, King Fahad Medical City, Riyadh, Saudi Arabia; ^4^Tadaw Medical Complex and Day Surgery Center, Riyadh, Saudi Arabia; ^5^Department of Gastroenterology and Hepatology, King Fahad Medical City, Riyadh, Saudi Arabia; ^6^Department of Medical Imaging Administration, King Fahad Medical City, Riyadh, Saudi Arabia; ^7^Department of Medicine, College of Medicine, King Saud University, Riyadh, Saudi Arabia

**Keywords:** type 2 diabetes, non-alcoholic fatty liver disease, carbohydrate, diet, nutrition

## Abstract

**Background:**

Non-alcoholic fatty liver disease (NAFLD) is an overlooked complication of type 2 diabetes (T2D). Current recommendations for the management of NAFLD are mainly focused on weight reduction, overlooking the role of macronutrient composition. Although dietary carbohydrates play a major role in intrahepatic fat synthesis, their association with the progression of liver steatosis has not been fully investigated in patients with T2D.

**Aim:**

To investigate the association between higher carbohydrate intake and the presence of liver steatosis in patients with T2D.

**Methods:**

This cross-sectional study included men and women aged 18–60 years diagnosed with T2D. Anthropometric measurements, hepatic steatosis assessment using the controlled attenuation parameter (CAP), blood samples, and dietary data were analyzed. Participants were divided into two groups: NAFLD and NAFLD-free. A two-sample *t*-test was used to evaluate the differences between the two groups. Stepwise multiple linear regression models adjusted for potential confounders were used to determine the association between CAP values and higher carbohydrate intake.

**Results:**

In total, 358 participants were included. NAFLD was present in 79.3% of the participants. Body mass index, waist circumference, ALT, HbA1c, and triglycerides showed direct, while HDL-Cholesterol revealed inverse associations with CAP values. No significant relationship was found between carbohydrate intake and steatosis in the total study sample; however, multiple linear regression analysis revealed a significant relationship between carbohydrate intake and CAP values in patients aged ≤50 years.

**Conclusion:**

In patients with T2D, higher carbohydrate intake was associated with liver steatosis in those aged 50 years and below. Further studies are required to confirm the causality between carbohydrate intake and liver steatosis.

## Introduction

Type 2 diabetes (T2D) is one of the fastest-growing diseases worldwide, representing a great healthcare challenge at present (1). Recent reports have demonstrated that T2D is rising in parallel with the increasing incidence of non-alcoholic fatty liver disease (NAFLD) (2), an overlooked complication of T2D. NAFLD is the most widespread form of chronic liver disease (2) and represents a spectrum of chronic hepatic diseases caused by fat accumulation in more than 5% of hepatocytes in the absence of significant alcohol abuse (3, 4). The global pooled prevalence of NAFLD in T2D is 59.67% as reported in a meta-analysis (5). In Saudi Arabia (SA), the prevalence of NAFLD was estimated at 72.8% among patients with T2D (6).

In patients with T2D, chronic increased levels of glucose, insulin, and free fatty acids contribute to resistance to insulin-stimulated glucose uptake in skeletal muscles and adipose tissue, in addition to resistance to the insulin-mediated suppression of lipolysis in adipose tissue (7). Moreover, elevated blood glucose concentrations lead to increased glucose uptake by the liver, resulting in increased conversion of glucose to fatty acids *via de novo* lipogenesis. As a result of the high blood glucose and free fatty acid concentrations, and to prevent the lipotoxicity of free fatty acids, fat is mildly accumulated in the liver as an adaptive response. As free fatty acids continue to flow into the liver, hepatic intracellular triglycerides will increase, leading to excessive hepatic lipid accumulation. Unlike healthy individuals, the exportation of hepatic fat by very low-density lipoprotein (VLDL) is either insufficient or impaired in patients with NAFLD and/or T2D (7, 8). Patients with T2D usually experience increased liver lipogenesis (9, 10), along with reduction in fatty acid oxidation (11) and triglycerides secretion through VLDL (12). A patient with T2D who has developed NAFLD is exposed to many complications, such as increased microvascular comorbidities of T2D (13, 14), increased risk of cardiovascular events (15), and the progression of the fatty liver into more severe and fatal conditions such as fibrosis, cirrhosis, and hepatocellular carcinoma. These adverse health conditions pose an extra burden on the national healthcare systems, especially with the increasing rates of T2D in SA (16, 17).

The current literature suggests weight reduction as a primary approach to managing NAFLD (4, 18, 19). However, these recommendations are mainly focused on calorie deficit, overlooking the role of dietary macronutrient composition. Macronutrient composition, and more specifically carbohydrate content, may modulate the success of intrahepatic fat reduction, as suggested by multiple studies (20–22). Theoretically, increased intake of dietary carbohydrates is known to be a strong contributor to the development of NAFLD, since they stimulate *de novo* lipogenesis and induce the synthesis of intrahepatic fat (23, 24). However, research in this area is limited and evidence is still scarce, which may be attributed to methodological limitations in collecting dietary data and achieving a representative sample size (25). Moreover, limited data is available to support the role of carbohydrate intake on the progression of NAFLD in patients with T2D. Knowledge achieved in this area will sufficiently contribute to the current dietary guidelines for patients with T2D, thus helping prevent NAFLD and its advanced stages. Therefore, in this cross-sectional study, we aim to investigate the relationship between carbohydrate intake and the presence of hepatic steatosis in patients diagnosed with T2D. We hypothesize that higher carbohydrate intake is associated with hepatic steatosis in patients diagnosed with T2D.

## Materials and methods

### Study design

This is a cross-sectional analysis conducted on a sub-sample randomly selected from the CORDIAL cohort study (non-alcoholic fatty liver disease in a Saudi Cohort with type 2 diabetes mellitus) at the Obesity Research Center, King Saud University, Riyadh, Saudi Arabia. Details on the study have been published previously (26). In brief, the CORDIAL study is a large ongoing prospective cohort started in 2015 that aims to identify the history of hepatic steatosis in patients with T2D over 10 years, with 1,000 participants currently recruited.

The present study was focused on analyzing anthropometric measurements, liver imaging (FibroScan^®^), blood samples, and completing and analyzing dietary data for recruited participants to explore associations between carbohydrate intake and the presence of liver steatosis in patients with T2D. Signed consent forms were collected from each participant prior to their inclusion in the study and participants had the right to withdraw at any time. The study was approved by the Local Research Ethics Committee in King Fahad Medical City (IRB 12-344).

### Sample size

The sample size was calculated based on local data (6, 27) to determine the prevalence of NAFLD among patients with T2D with a two-sided significance level of 5% and a confidence level of 95%. The required sample size was 368 participants. Considering a 10% dropout rate, the final sample size was approximately 405 participants.

### Study participants

**FIGURE 1:**
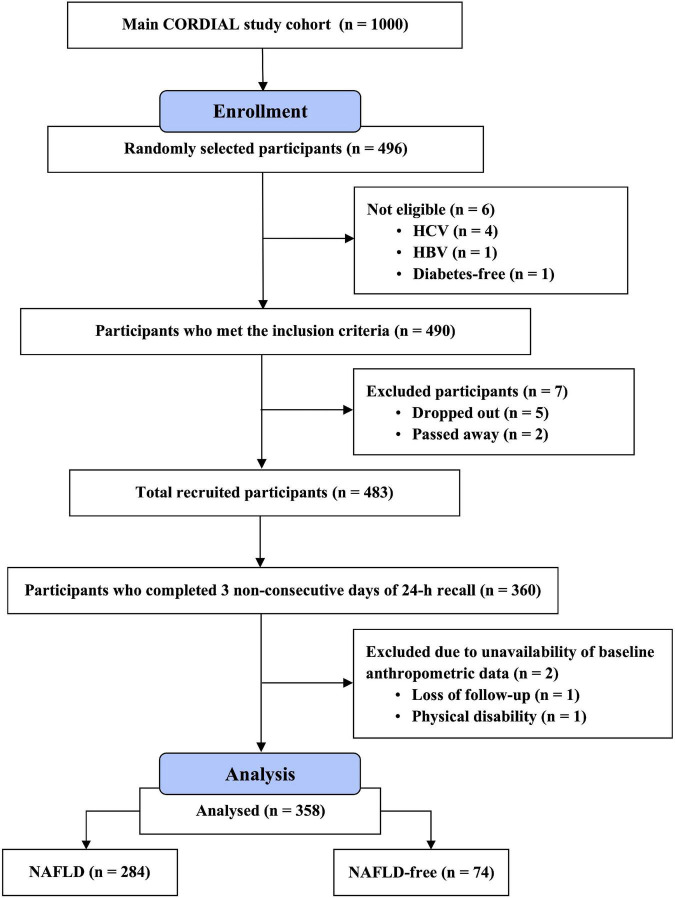
A flow chart of the recruitment process.

The CORDIAL study included men and women aged 18–60 years and diagnosed with T2D. Subjects were excluded if they showed evidence of hepatic decompensation, or if they have preexisting hepatocellular carcinoma, causes of fatty liver other than NAFLD, or a presence of significant alcohol intake (i.e., daily intake of ≥30 g for men and ≥20 g for women). For the current analyses, we excluded participants who did not complete three non-consecutive days of the 24-h dietary recall or had missing liver imaging data. The recruitment process is illustrated in Figure 1.

### Dietary data

Dietary data were assessed using a detailed 24-h recall assessment method for three non-consecutive days, two on weekdays and one on weekends. The 24-h recall data were collected by trained clinical dietitians utilizing the United States Department of Agriculture 5-step multiple-pass technique (28, 29). Owing to the COVID-19 restrictions, the protocol was adjusted to one dietary assessment conducted in an in-person visit and two by phone.

During the dietary assessment, three-dimensional measurement aids (such as food models and household measures) and two-dimensional measurement aids (such as food photographs) were used to estimate portion sizes of consumed food. Moreover, participants were provided a brochure that contains photographs and descriptions of different portion sizes and household measures to be utilized in phone interviews. Most of the two-dimensional measurement aids were obtained from the Nutritional Assessment Guide for Saudi Arabia (30), which contains comprehensive information on different local dishes displayed in various amounts and sizes. All dietary data were transformed into metric units and analyzed using ESHA’s Food Processor^®^ Nutrition Analysis software (ESHA Research, OR, USA) (31) to estimate macronutrient compositions. Saudi local dishes that were not available in ESHA’s database were entered as separate ingredients depending on the original recipe, using the Nutritional Assessment Guide for Saudi Arabia. In case a food item was unavailable, this was added manually if the nutritional information of that food was available, or the most similar available food item was used. Trained dietitians followed a study-specific operational manual and a standard protocol for data coding and entry.

### Liver steatosis assessment

To detect liver steatosis, a controlled attenuation parameter (CAP) test using FibroScan^®^ (Echosens Ltd., Paris, France) was conducted by a trained specialist. CAP is a valid, non-invasive measuring method that is used to diagnose and quantify hepatic steatosis. It can provide higher accuracy in diagnosing and assessing steatosis compared to circulating biomarkers or ultrasonography (32). Hepatic steatosis was determined based on FibroScan^®^ CAP cutoff values that range from S0, which indicates no steatosis, to S3, indicating severe steatosis. Steatosis stages were defined as follows: (i) S0 for no steatosis (CAP values less than 248 dB/m); (ii) S1 for mild steatosis (CAP values from 248 to <268 dB/m, indicating ≥10% of hepatocytes with fat); (iii) S2 for moderate steatosis (CAP values from 268 to <280 dB/m, indicating ≥33% hepatocytes with fat); (iv) S3 for severe steatosis (CAP values equal to 280 dB/m or more, indicating ≥66% hepatocytes with fat) (33).

### Anthropometric and biochemical measurements

Anthropometric parameters [height, weight, and waist circumference (WC)] were recorded once, using a standard protocol, with participants wearing light clothing and no shoes. Body mass index (BMI) was calculated as kg/m^2^. All blood samples were collected after overnight fasting, well-contained and stored in the main study site. Blood samples were collected mainly to measure liver enzymes, lipid profile, and glycated hemoglobin (HbA1c). Anthropometrics and blood samples were collected by a certified nurse. Serum lipids and liver enzymes were measured using Abbott – Architect Plus, a clinical chemistry autoanalyzer (Abbott, Abbott Park, IL, USA). Glycated hemoglobin (HbA1c) determination was performed using D-100^®^, a high-performance liquid chromatography analyzer (Bio-Rad Laboratories, Hercules, CA, USA).

### Statistical analysis

A normality test was performed for all variables and data were checked for outliers and data transfer errors. All included data were normally distributed. Participants were divided into two groups, NAFLD and NAFLD-free, and a two-sample *t*-test was used to evaluate the differences between the two groups. Continuous data were presented as mean ± standard deviation (SD) and categorical data were presented as frequencies and percentages.

Pearson’s correlation test was used to measure the association between continuous variables. Moreover, participants were stratified into subgroups based on steatosis severity (S0–S3). Analysis of variance (ANOVA) and χ^2^ tests were used to analyze differences between continuous and categorical variables, and Tukey’s *post-hoc* test was performed to detect the significant differences between groups.

We used stepwise multiple linear regression models adjusted for potential confounders to determine associations with CAP values for each 2 SD higher carbohydrate intake. Model 1 was adjusted for age and sex. Model 2 was additionally adjusted for demographic data (marital status, education, employment, and income) and smoking. Model 3 was additionally adjusted for BMI. Model 4 is model 2 adjusted for LDL-cholesterol (LDL-C) and HDL-cholesterol (HDL-C). Finally, model 5 is model 2 adjusted for triglycerides. Additionally, we evaluated potential effect modifications by age, sex, BMI, and WC. *P*-values of <0.05 were considered statistically significant for all analyses. Logistic regression analysis was also conducted to investigate the association between carbohydrate intake and the presence/absence of NAFLD, using the previously defined models. Moreover, receiver operating characteristic (ROC) curve analysis was performed to assess the models. All statistical analyses were performed using the Statistical Package for Social Science (IBM SPSS^®^) version 23.0 (SPSS Inc., IBM, Armonk, NY, USA).

## Results

### Baseline characteristics

A total of 358 participants were included in the current study. Descriptive characteristics of the study sample are presented in Table 1. All variables were normally distributed. Approximately 79% of participants were diagnosed with NAFLD, of which 97.2% were overweight or obese. BMI was significantly higher in the NAFLD group (33.4 ± 5.1 kg/m^2^) compared to the NAFLD-free group (30.9 ± 6.3 kg/m^2^, *P* < 0.001). Similarly, the mean WC was higher in the NAFLD group (107.1 ± 13.0 cm) compared to the NAFLD-free group (103.3 ± 12.9 cm, *P* = 0.03). Significant differences were found between the NAFLD and NAFLD-free groups in ALT (25.6 ± 14.1 U/L vs. 21.1 ± 8.9 U/L, *P* = 0.009), HDL-C (1.1 ± 0.3 mmol/L vs. 1.2 ± 0.3 mmol/L, *P* = 0.04), and triglycerides (1.6 ± 0.8 mmol/L vs. 1.3 ± 0.6 mmol/L, *P* = 0.001) (Table 1).

**TABLE 1:** Baseline characteristics of the study sample stratified by the presence of non-alcoholic fatty liver disease (NAFLD), *n* = 358.

Characteristics	Total sample (*n* = 358)	NAFLD (CAP ≥ 248) (*n* = 284)	NAFLD-free (CAP < 248) (*n* = 74)	*P*-value
Age (years)	49.8 ± 7.5 (range: 25–60)	49.6 ± 7.5	50.7 ± 7.4	0.274
**Sex**				
Female	175 (48.9%)	147 (51.8%)	28 (37.8%)	0.033
Male	183 (51.1%)	137 (48.2%)	46 (62.2%)	
**Anthropometric measurements**				
BMI (kg/m^2^)	32.9 ± 5.5	33.4 ± 5.1	30.9 ± 6.3	< 0.001
Lean (<25)	22 (6.1%)	8 (2.8%)	14 (18.9%)	
Overweight (≥25)	336 (93.9%)	276 (97.2%)	60 (81.1%)	< 0.001
Waist circumference	106.3 ± 13	107.06 ± 12.96	103.25 ± 12.89	0.025
Men	107.3 ± 13.6	109.1 ± 13.2	102.1 ± 13.4	0.002
Women	105.2 ± 12.4	105.2 ± 12.5	105.2 ± 12	0.996
**Liver steatosis**				
CAP (dB/m)	295.9 ± 58.9	318.1 ± 41	210.5 ± 33.4	< 0.001
**Biochemistry**				
ALT (U/L)	24.7 ± 13.3	25.6 ± 14.1	21.08 ± 8.88	0.009
AST (U/L)	20.6 ± 8.8	20.9 ± 9.3	19.6 ± 6.3	0.273
GGT (U/L)	33 ± 32.8	31.5 ± 24.7	38.9 ± 6.3	0.108
Total cholesterol (mmol/L)	4.3 ± 1.2	4.3 ± 1.2	4.2 ± 1.1	0.457
HDL-C (mmol/L)	1.1 ± 0.3	1.1 ± 0.3	1.2 ± 0.3	0.044
LDL-C (mmol/L)	2.7 ± 0.9	2.7 ± 0.9	2.6 ± 0.9	0.326
Triglycerides (mmol/L)	1.6 ± 0.8	1.6 ± 0.8	1.3 ± 0.6	0.001
HbA1c (%)	8.3 ± 1.9	8.3 ± 1.9	8 ± 1.9	0.146
**Dietary intake**				
Carbohydrate (%energy/day)	57.8 ± 8.4	58.1 ± 8	56.9 ± 8.6	0.237
Protein (%energy/day)	17 ± 3.9	16.9 ± 3.9	17.4 ± 4.2	0.282
Fat (%energy/day)	25.3 ± 7	25 ± 6.5	25.7 ± 7.3	0.418
%Saturated fat	6.8 ± 2.8	6.7 ± 2.4	6.8 ± 2.5	0.626
%Monounsaturated fat	6.5 ± 3	6.4 ± 2.9	6.8 ± 3.4	0.304
%Polyunsaturated fat	3.1 ± 1.9	3.1 ± 1.9	3.1 ± 2.1	0.978
%Trans fat	0.1 ± 0.2	0.1 ± 0.1	0.2 ± 0.2	0.086
%Other fat	8.8 ± 4.2	8.7 ± 4	8.9 ± 4.3	0.721
Dietary cholesterol (mg/1000 cal)	109.1 ± 58.7	107.1 ± 56.2	115.7 ± 64.1	0.252
Energy (kcal/day)	1737.4 ± 661.8	1747.6 ± 673.9	1651.9 ± 580.5	0.265
Fiber (g/1000 kcal)	12.1 ± 4.3	11.9 ± 3.8	12.5 ± 4.8	0.265
Sugar (g/1000 kcal)	27.4 ± 13.5	27.1 ± 13	28.4 ± 14.4	0.427

Data presented as mean ± SD for continuous variables and *n* (%) for categorical variables; *p* < 0.05 is considered significant. NAFLD, non-alcoholic fatty liver disease; CAP, controlled attenuation parameter; ALT, alanine transaminase; AST, aspartate aminotransferase; GGT, gamma-glutamyl transferase; HDL-C, high-density lipoprotein cholesterol; LDL-C, low-density lipoprotein cholesterol; HbA1c, glycated hemoglobin.

### Associations between hepatic steatosis and other parameters

In the total study sample, CAP values were significantly and directly correlated with BMI (*r* = 0.2, *P* < 0.001), WC (*r* = 0.2, *P* < 0.001), and being overweight (*r* = 0.3, *P* < 0.001). CAP values were significantly correlated with higher ALT levels (*r* = 0.3, *P* < 0.001), triglyceride levels (*r* = 0.2, *P* = 0.003), and lower HDL-C levels (*r* = −0.2, *P* = 0.002) in the total study sample. Moreover, greater HbA1c levels were significantly correlated with higher CAP values (*r* = 0.1, *P* = 0.02). No significant associations were found between NAFLD and nutrient intake (Tables 1, 2).

**TABLE 2:** Analysis of variance between hepatic steatosis severity and weight, biochemical, and dietary data, *n* = 358.

Factors	S0 (*n* = 74)	S1 (*n* = 34)	S2 (*n* = 23)	S3 (*n* = 227)	ANOVA *P*-value
BMI	30.9 ± 6.3^c^	32.8 ± 3.9	32.5 ± 4.4	33.6 ± 5.3	0.003
Lean	14 (18.9%)^a, b, c^	0	1 (4.3%)	7 (3.1%)	< 0.001
Overweight	60 (81.1%)^a, b, c^	31 (100%)	22 (95.7%)	220 (96.9%)	< 0.001
Waist circumference	103.3 ± 12.9^c^	103 ± 10.6	102.7 ± 11.5	108.1 ± 13.2	0.007
AST (U/L)	19.6 ± 6.3	17.6 ± 4.9	18.8 ± 6	21.5 ± 9.9	0.059
ALT (U/L)	21.1 ± 8.9^c^	19.2 ± 8	20.6 ± 8.5	27.0 ± 14.9	< 0.001
GGT (U/L)	38.9 ± 53.6	32 ± 41	24.5 ± 14.4	32.1 ± 22.3	0.331
Total cholesterol (mmol/L)	4.2 ± 1.1	4.3 ± 1.3	4.3 ± 0.9	4.3 ± 1.2	0.895
HDL-C (mmol/L)	1.2 ± 0.3	1.2 ± 0.3	1.2 ± 0.4	1.1 ± 0.3	0.027
LDL-C (mmol/L)	2.6 ± 0.9	2.7 ± 1.1	2.6 ± 0.9	2.7 ± 0.9	0.649
Triglycerides (mmol/L)	1.3 ± 0.6^c^	1.5 ± 0.8	1.7 ± 0.9	1.7 ± 0.8	0.008
HbA1c (%)	8 ± 1.9	8.2 ± 2.2	8 ± 1.6	8.4 ± 1.8	0.359
Carbohydrate (%energy/day)	56.9 ± 8.6	56.8 ± 7.2	59.7 ± 6.7	58.2 ± 8.3	0.372
Protein (%energy/day)	17.4 ± 4.1	17.1 ± 4.2	16.3 ± 3.5	16.9 ± 3.9	0.627
Fat (%energy/day)	25.7 ± 7.3	26.1 ± 6.4	24 ± 5.1	25 ± 6.6	0.548
%Saturated fat	6.8 ± 2.5	7.4 ± 3.0	6 ± 1.5	6.6 ± 2.4	0.165
%Monounsaturated fat	6.8 ± 3.2	7 ± 3.3	5.6 ± 2	6.4 ± 2.9	0.256
%Polyunsaturated fat	3.1 ± 2.1	3.2 ± 2.1	3.1 ± 2.2	3.1 ± 1.8	0.993
%Trans fat	0.2 ± 0.2	0.2 ± 0.2	0.1 ± 0.1	0.1 ± 0.1	0.201
%Other fat	8.9 ± 4.4	8.4 ± 4.0	9.2 ± 4	8.7 ± 4	0.871
Dietary cholesterol (mg/1000 kcal)	115.7 ± 64.1	110.8 ± 54.5	104.1 ± 47	106.8 ± 57.5	0.680
Energy (kcal/day)	1651.9 ± 580.5	1780.5 ± 544.0	1522.7 ± 614.4	1765.4 ± 695	0.241
Fiber (g/1000 kcal)	12.5 ± 4.8	11.7 ± 3.1	12.5 ± 4.3	11.9 ± 3.9	0.614
Sugar (g/1000 kcal)	28.4 ± 14.4	27.8 ± 11.7	25.1 ± 12.3	27.2 ± 13.3	0.737

Data presented as mean ± SD for continuous variables and *n* (%) for categorical variables; *p* < 0.05 is considered significant. NAFLD, non-alcoholic fatty liver disease; CAP, controlled attenuation parameter; ALT, alanine transaminase; AST, aspartate aminotransferase; GGT, gamma-glutamyl transferase; HDL-C, high-density lipoprotein cholesterol; LDL-C, low-density lipoprotein cholesterol; HbA1c, glycated hemoglobin.

^a^Significant difference between S0 and S1.

^b^Significant difference between S0 and S2.

^c^Significant difference between S0 and S3.

### Associations between hepatic steatosis severity, blood biochemistry, and body mass index

When the means of steatosis severity groups were compared to each other, the ANOVA test revealed significant differences in BMI, WC, overweight, ALT, triglycerides, and HDL-C. In Tukey’s *post-hoc* analysis, participants with no steatosis have significantly lower BMI, WC, ALT, and triglycerides compared to the severe steatosis group (*P* = 0.001, *P* = 0.03, *P* = 0.004, and *P* = 0.005, respectively) (Table 2).

### Associations between carbohydrate intake and controlled attenuation parameter values

Linear regression analysis showed no statistically significant association between carbohydrate intake and CAP in all models for the total study sample (Supplementary Table 1).

Test of interaction revealed a significant effect of age as a modifier; therefore, we stratified the study sample by median age (51 years) and repeated the regression analysis (Supplementary Table 1). In participants aged 50 years and below, carbohydrate intake was significantly associated with CAP in model 3 (*B* = 1.249, *P* = 0.034) and model 4 (*B* = 1.400, *P* = 0.025) (Table 3). No significant associations were observed in the other age group (>50 years) (Supplementary Table 1). In the logistic regression analysis, carbohydrate intake did not have any significant contribution in all models (Supplementary Table 2). In the total sample, ROC curve analysis revealed significant results in models 2–5 (*P* < 0.001) (Supplementary Figure 1 and Supplementary Table 3). Similar findings were observed in participants aged ≤50 and >50 years (Supplementary Figures 2, 3 and Supplementary Table 3).

**TABLE 3:** Stepwise multiple linear regression analysis between CAP values and two standard deviation higher carbohydrate intake in participants aged 50 years and below, *n* = 163.

Model	Unstandardized coefficient		Standardized coefficient	*t*	*P*-value	95% CI	
	*B*	Standard error				Lower bound	Upper bound
**Model 3**							
Carbohydrate intake (%energy/day)	1.249	0.583	0.171	2.143	0.034	0.097	2.401
Sex	18.100	11.450	0.154	1.581	0.116	–4.531	40.731
Marital status	–10.804	6.118	–0.137	–1.766	0.079	–22.895	1.287
Level of education	3.830	2.570	0.120	1.490	0.138	–1.249	8.909
Employment	–7.341	3.108	–0.211	–2.362	0.020	–13.484	–1.197
Average family income	0.378	2.963	0.010	0.127	0.899	–5.478	6.233
Smoking	8.802	17.220	0.042	0.511	0.610	–25.232	42.835
BMI (kg/m^2^)	3.243	0.842	0.326	3.852	< 0.001	1.579	4.907
**Model 4**							
Carbohydrate intake (%energy/day)	1.400	0.618	0.191	2.267	0.025	0.180	2.621
Sex	1.750	12.533	0.015	0.140	0.889	–23.024	26.525
Marital status	–10.843	6.267	–0.137	–1.730	0.086	–23.232	1.545
Level of education	4.157	2.618	0.131	1.588	0.115	–1.018	9.332
Employment	–4.748	3.147	–0.136	–1.509	0.134	–10.969	1.473
Average family income	–0.172	3.022	–0.005	–0.057	0.955	–6.145	5.800
Smoking	4.932	17.707	0.023	0.279	0.781	–30.069	39.934
LDL-C (mmol/L)	11.074	4.795	0.187	2.309	0.022	1.595	20.553
HDL-C (mmol/L)	–38.685	18.448	–0.185	–2.097	0.038	–75.152	–2.219

*p* < 0.05 is considered significant. CAP, controlled attenuation parameter; CI, confidence interval; BMI, body mass index; HDL-C, high-density lipoprotein cholesterol; LDL-C, low-density lipoprotein cholesterol.

## Discussion

To the best of our knowledge, this is the first national study investigating carbohydrate intake in relation to CAP-confirmed NAFLD in participants diagnosed with T2D. In this study, hepatic steatosis was associated with higher carbohydrate consumption in participants aged ≤50 years in the linear regression model 3, which was adjusted for sex, demographic data, smoking, and BMI, and model 4, which was adjusted for sex, demographic data, smoking, LDL-C, and HDL-C. Moreover, we found significant associations between hepatic steatosis and higher BMI, WC, triglycerides, ALT, HbA1c, and lower HDL-C in the total study sample.

### Frequency of non-alcoholic fatty liver disease among patients with type 2 diabetes

Non-alcoholic fatty liver disease was present in 79.3% of the study participants. Similarly, a study conducted in Abha city (2018) showed that NAFLD was present in 72.8% of 245 participants previously diagnosed with T2D (6). Another study in Jeddah (2003) showed that the prevalence of NAFLD among 116 patients with T2D was 55% (34). Both SA studies used abdominal ultrasound examination to diagnose NAFLD, whereas FibroScan^®^ was used in our study. Globally, the pooled prevalence of NAFLD in patients with T2D in a meta-analysis that included 24 studies involving 35,599 patients with T2D was 59.67% (5). Another meta-analysis of 17 studies involving 10,897 patients with T2D reported an overall NAFLD prevalence of 54% (35). In both reports, the highest prevalence rates were reported in Romania (87.1%), India (87%), and Italy (75%). In our study, the detected frequency of NAFLD among patients with T2D is thought to be in line with the recent local and global data.

### Carbohydrate consumption and its association with hepatic steatosis

Although participants in the NAFLD group consumed slightly more carbohydrates than the NAFLD-free group, this was not statistically significant. Moreover, the results of our study showed no significant association between carbohydrate intake and CAP values and with steatosis stages in the total study sample. Several studies found otherwise (21, 36, 37); less carbohydrate intake was associated with lower intrahepatic fat. It is noteworthy that some of these studies used absolute consumption in grams instead of energy-adjusted intake (21).

Interestingly, linear regression models revealed a significant association between carbohydrate intake and CAP in participants aged ≤50 years, after adjusting for demographic variables, smoking, BMI, LDL-C, and HDL-C. The underlying mechanism remains unclear; however, this may suggest that younger individuals with T2D are more likely to benefit from lower carbohydrate intake to prevent the development of NAFLD. Similar to our findings, the Rotterdam study did not find an association between steatosis and carbohydrate intake in their predominantly older study sample (38). Although the studies that found a desirable effect of less carbohydrate consumption on liver steatosis were not focused on those diagnosed with T2D, it is noticeable that the mean age of the included participants in some of these studies was comparable to the mean age of our younger group (43.2 ± 5.8 years) (20, 36, 39).

### Clinical and biochemical parameters and their relation to non-alcoholic fatty liver disease

In our study, higher weight was associated with the presence of hepatic steatosis in patients with T2D. These findings contribute to the accumulating evidence that NAFLD is associated with increased body weight and central obesity (40–43). According to a meta-analysis of 16 studies, BMI and WC were both independently associated with NAFLD (44). Individuals with central obesity had a higher risk of NAFLD than individuals with general obesity (44, 45), as central obesity may interrupt the secretion of adipose tissue-derived adipokines, leading to an increase in harmful and a decrease in protective adipocytokines (46, 47), which may accelerate the occurrence of NAFLD (48, 49).

The present study showed that HbA1c was significantly associated with CAP values. This suggests that poor diabetes control could be a significant contributor to the development of liver steatosis. It has been suggested that in T2D, hyperglycemia induces the generation of oxidative stress markers and inflammatory mediators leading to cell dysfunction (50, 51). In patients with both T2D and NAFLD, the emission of inflammatory mediators takes place in an early stage of the disease prior to liver damage. Therefore, to prevent further progression of NAFLD into its more severe stages, more focus should be given to monitoring blood glucose levels, HbA1c, and lipid profile, in addition to encouraging a healthy lifestyle (51).

We found that liver steatosis in patients with T2D was correlated with dyslipidemia (i.e., high triglycerides and low HDL-C), which was observed in previous studies as well (51, 52). It is known that T2D-induced hyperglycemia is likely to enhance the pathogenicity of NAFLD by inducing dyslipidemia (53). Furthermore, increased serum triglyceride level is associated with insulin resistance, which also leads to hepatocyte fat deposition. With respect to liver enzymes, ALT was correlated with hepatic steatosis, which is comparable to previous studies (40, 54). Ultrasonography-detected NAFLD was the most common cause of abnormal liver biochemistry, according to a large prospective cohort study from the United Kingdom (55). In this study, participants diagnosed with NAFLD had significantly higher WC, triglycerides, and HbA1c and lower HDL-C, which are the characteristics of metabolic syndrome (56). NAFLD is currently considered a hepatic manifestation of metabolic syndrome in patients with T2D (57, 58). The links between NAFLD, metabolic syndrome, and T2D are probable due to the shared pathogenic factors (59).

This is the first national study to investigate carbohydrate intake in relation to CAP-confirmed NAFLD in participants diagnosed with T2D in Saudi Arabia and the first to identify age as a potential effect modifier. A key strength of our study is the use of a non-invasive diagnostic tool to assess liver steatosis, the CAP. Moreover, we performed a comprehensive, detailed dietary assessment using the best available method (24-h recall), which was conducted on three non-consecutive days to allow for the correction of within-subject variability in nutrient intake (60). Furthermore, dietary data were analyzed using a validated food analysis software, with reliance on local food guides to estimate the nutritional values of the traditional dishes. On the contrary, this study has potential limitations that need to be considered. The cross-sectional nature of the study makes it difficult to infer causality. Further limitations include residual confounding and measurement errors and the possibility of misreporting the actual dietary intake, which is a common limitation of the 24-h recall method.

## Conclusion

In adult patients who are diagnosed with T2D, the results of our study support our hypothesis that higher carbohydrate intake is associated with liver steatosis in patients aged ≤50 years. Further studies are needed to confirm causality and help make dietary recommendations for patients with T2D and/or NAFLD. Future studies are needed to investigate whether younger individuals with T2D are more likely to benefit from lower carbohydrate intake to prevent the development of NAFLD.

## Data availability statement

The datasets presented in this article are not readily available. Requests to access the datasets should be directed to corresponding author.

## Ethics statement

The studies involving human participants were reviewed and approved by the Local Research Ethics Committee in King Fahad Medical City, Riyadh, Saudi Arabia. The participants provided their written informed consent to participate in this study.

## Author contributions

NA, GA, and AAA contributed to the design of the study. AAA, SS, ANA, ASA, and AMA contributed to participant recruitment. NA and HA collected and analyzed the dietary data. MK collected the clinical data. NA and GA wrote the manuscript. All authors participated in interpretation of the data and reviewed and approved the final manuscript.

## References

[1] Unnikrishnan R Pradeepa R Joshi SR Mohan V. Type 2 diabetes: demystifying the global epidemic. *Diabetes.* (2017) 66:1432. 10.2337/db16-0766 28533294

[2] Calzadilla Bertot L Adams LA. The natural course of non-alcoholic fatty liver disease. *Int J Mol Sci.* (2016) 17:774. 10.3390/ijms17050774 27213358 PMC4881593

[3] Benedict M Zhang X. Non-alcoholic fatty liver disease: an expanded review. *World J Hepatol.* (2017) 9:715–32. 10.4254/wjh.v9.i16.715 28652891 PMC5468341

[4] Alswat KA Fallatah HI Al-Judaibi B Elsiesy HA Al-Hamoudi WK Qutub AN Position statement on the diagnosis and management of non-alcoholic fatty liver disease. *Saudi Med J.* (2019) 40:531–40. 10.15537/smj.2019.6.23980 31219486 PMC6778754

[5] Dai W Ye L Liu A Wen SW Deng J Wu X Prevalence of nonalcoholic fatty liver disease in patients with type 2 diabetes mellitus: a meta-analysis. *Medicine.* (2017) 96:e8179. 10.1097/MD.0000000000008179 28953675 PMC5626318

[6] Alsabaani AA Mahfouz AA Awadalla NJ Musa MJ Al Humayed SM. Non-alcoholic fatty liver disease among type-2 diabetes mellitus patients in Abha City, South Western Saudi Arabia. *Int J Environ Res Public Health.* (2018) 15:2521. 10.3390/ijerph15112521 30423871 PMC6266142

[7] Zivkovic AM German JB Sanyal AJ. Comparative review of diets for the metabolic syndrome: implications for nonalcoholic fatty liver disease. *Am J Clin Nutr.* (2007) 86:285–300. 10.1093/ajcn/86.2.285 17684197

[8] Xia MF Bian H Gao X. Nafld and diabetes: two sides of the same coin? Rationale for gene-based personalized nafld treatment. *Front Pharmacol.* (2019) 10:877. 10.3389/fphar.2019.00877 31447675 PMC6691129

[9] Tian J Goldstein JL Brown MS. Insulin induction of srebp-1c in rodent liver requires Lxrα-C/Ebpβ complex. *Proc Natl Acad Sci.* (2016) 113:8182–7. 10.1073/pnas.1608987113 27382175 PMC4961151

[10] Linden AG Li S Choi HY Fang F Fukasawa M Uyeda K Interplay between Chrebp and Srebp-1c coordinates postprandial glycolysis and lipogenesis in livers of mice. *J Lipid Res.* (2018) 59:475–87. 10.1194/jlr.M081836 29335275 PMC5832931

[11] Schmid AI Szendroedi J Chmelik M Krššák M Moser E Roden M. Liver atp synthesis is lower and relates to insulin sensitivity in patients with type 2 diabetes. *Diabetes Care.* (2011) 34:448–53. 10.2337/dc10-1076 21216854 PMC3024365

[12] Kamagate A Dong HH. Foxo1 integrates insulin signaling to Vldl production. *Cell Cycle.* (2008) 7:3162–70. 10.4161/cc.7.20.6882 18927507 PMC2664837

[13] Targher G Lonardo A Byrne CD. Nonalcoholic fatty liver disease and chronic vascular complications of diabetes mellitus. *Nat Rev Endocrinol.* (2018) 14:99–114. 10.1038/nrendo.2017.173 29286050

[14] Ix JH Sharma K. mechanisms linking obesity, chronic kidney disease, and fatty liver disease: the roles of Fetuin-a, Adiponectin, and Ampk. *J Am Soc Nephrol.* (2010) 21:406–12. 10.1681/asn.2009080820 20150538 PMC4473254

[15] Targher G Bertolini L Rodella S Tessari R Zenari L Lippi G Nonalcoholic fatty liver disease is independently associated with an increased incidence of cardiovascular events in type 2 diabetic patients. *Diabetes Care.* (2007) 30:2119–21. 10.2337/dc07-0349 17519430

[16] Alhowaish AK. Economic costs of diabetes in Saudi Arabia. *J Fam Commun Med.* (2013) 20:1–7. 10.4103/2230-8229.108174 23723724 PMC3663158

[17] Robert AA Al Dawish AM Braham R Musallam AM Al Hayek AA Al Kahtany HN. Type 2 diabetes mellitus in Saudi Arabia: major challenges and possible solutions. *Curr Diabetes Rev.* (2017) 13:59–64. 10.2174/1573399812666160126142605 26813972

[18] Koutoukidis DA Astbury NM Tudor KE Morris E Henry JA Noreik M Association of weight loss interventions with changes in biomarkers of nonalcoholic fatty liver disease: a systematic review and meta-analysis. *JAMA Intern Med.* (2019) 179:1262–71. 10.1001/jamainternmed.2019.2248 31260026 PMC6604126

[19] Zelber-Sagi S Godos J Salomone F. Lifestyle changes for the treatment of nonalcoholic fatty liver disease: a review of observational studies and intervention trials. *Therap Adv Gastroenterol.* (2016) 9:392–407. 10.1177/1756283X16638830 27134667 PMC4830109

[20] Solga S Alkhuraishe AR Clark JM Torbenson M Greenwald A Diehl AM Dietary composition and nonalcoholic fatty liver disease. *Dig Dis Sci.* (2004) 49:1578–83. 10.1023/B:DDAS.0000043367.69470.b7 15573908

[21] Gonzalez C de Ledinghen V Vergniol J Foucher J Le Bail B Carlier S Hepatic steatosis, carbohydrate intake, and food quotient in patients with Nafld. *Int J Endocrinol.* (2013) 2013:428542. 10.1155/2013/428542 23737773 PMC3659479

[22] Sevastianova K Santos A Kotronen A Hakkarainen A Makkonen J Silander K Effect of short-term carbohydrate overfeeding and long-term weight loss on liver fat in overweight humans. *Am J Clin Nutr.* (2012) 96:727–34. 10.3945/ajcn.112.038695 22952180

[23] Worm N. Beyond body weight-loss: dietary strategies targeting intrahepatic fat in Nafld. *Nutrients.* (2020) 12:1316. 10.3390/nu12051316 PMC7284418 32384593

[24] Parks EJ. Dietary carbohydrate’s effects on lipogenesis and the relationship of lipogenesis to blood insulin and glucose concentrations. *Br J Nutr.* (2002) 87 (Suppl. 2):S247–53. 10.1079/BJNBJN/2002544 12088525

[25] Neuschwander-Tetri BA. Carbohydrate intake and nonalcoholic fatty liver disease. *Curr Opin Clin Nutr Metab Care.* (2013) 16:446–52. 10.1097/mco.0b013e328361c4d1 23657151

[26] Alfadda AA Sherbeeni SM Alqutub AN Aldosary AS Aldaghri NM Taylor-Robinson SD Transient elastography for the prevalence of non-alcoholic fatty liver disease in patients with type 2 diabetes: evidence from the cordial cohort study. *Saudi J Gastroenterol.* (2022) 28:426–33. 10.4103/sjg.sjg_73_22 35645140 PMC9843508

[27] Elmakki E Aqeely H Bani I Omer H Solan Y Taher A Nonalcoholic fatty liver disease (Nafld) in Saudi patients with T2dm in Jazan region: prevalence and associated factors. *Br J Med Med Res.* (2015) 5:872–9. 10.9734/bjmmr/2015/13077

[28] Conway JM Ingwersen LA Moshfegh AJ. Accuracy of dietary recall using the usda five-step multiple-pass method in men: an observational validation study. *J Am Diet Assoc.* (2004) 104:595–603. 10.1016/j.jada.2004.01.007 15054345

[29] Conway JM Ingwersen LA Vinyard BT Moshfegh AJ. Effectiveness of the Us department of agriculture 5-step multiple-pass method in assessing food intake in obese and nonobese women. *Am J Clin Nutr.* (2003) 77:1171–8. 10.1093/ajcn/77.5.1171 12716668

[30] Alfadda AA. *The nutritional assessment guide for Saudi Arabia.* Olaya: King Fahad National Library (2018).

[31] Bazzano LA He J Ogden LG Loria CM Vupputuri S Myers L Agreement on nutrient intake between the databases of the first national health and nutrition examination survey and the esha food processor. *Am J Epidemiol.* (2002) 156:78–85. 10.1093/aje/kwf003 12076891

[32] Sirli R Sporea I. Controlled attenuation parameter for quantification of steatosis: which cut-offs to use? *Can J Gastroenterol Hepatol.* (2021) 2021:6662760. 10.1155/2021/6662760 33834008 PMC8018863

[33] Mikolasevic I Orlic L Franjic N Hauser G Stimac D Milic S. Transient elastography (Fibroscan(^®^)) with controlled attenuation parameter in the assessment of liver steatosis and fibrosis in patients with nonalcoholic fatty liver disease – where do we stand? *World J Gastroenterol.* (2016) 22:7236–51. 10.3748/wjg.v22.i32.7236 27621571 PMC4997649

[34] Akbar DH Kawther AH. Nonalcoholic Fatty liver disease in saudi type 2 diabetic subjects attending a medical outpatient clinic: Prevalence and general characteristics. *Diabetes Care.* (2003) 26:3351–2. 10.2337/diacare.26.12.3351-a 14633828

[35] Amiri Dash Atan N Koushki M Motedayen M Dousti M Sayehmiri F Vafaee R Type 2 diabetes mellitus and non-alcoholic fatty liver disease: a systematic review and meta-analysis. *Gastroenterol Hepatol Bed Bench.* (2017) 10 (Suppl. 1):S1–7. 29511464 PMC5838173

[36] Browning JD Baker JA Rogers T Davis J Satapati S Burgess SC. Short-term weight loss and hepatic triglyceride reduction: evidence of a metabolic advantage with dietary carbohydrate restriction. *Am J Clin Nutr.* (2011) 93:1048–52. 10.3945/ajcn.110.007674 21367948 PMC3076656

[37] Ryan MC Itsiopoulos C Thodis T Ward G Trost N Hofferberth S The mediterranean diet improves hepatic steatosis and insulin sensitivity in individuals with non-alcoholic fatty liver disease. *J Hepatol.* (2013) 59:138–43. 10.1016/j.jhep.2013.02.012 23485520

[38] Alferink LJ Kiefte-de Jong JC Erler NS Veldt BJ Schoufour JD de Knegt RJ Association of dietary macronutrient composition and non-alcoholic fatty liver disease in an ageing population: the rotterdam study. *Gut*. (2019) 68:1088–98. 10.1136/gutjnl-2017-315940 30064987

[39] Haufe S Engeli S Kast P Bohnke J Utz W Haas V Randomized comparison of reduced fat and reduced carbohydrate hypocaloric diets on intrahepatic fat in overweight and obese human subjects. *Hepatology.* (2011) 53:1504–14. 10.1002/hep.24242 21400557

[40] Mansour-Ghanaei F Joukar F Mobaraki SN Mavaddati S Hassanipour S Sepehrimanesh M. Prevalence of non-alcoholic fatty liver disease in patients with diabetes mellitus, hyperlipidemia, obesity and polycystic ovary syndrome: a cross-sectional study in North of Iran. *Diabetes Metab Syndr.* (2019) 13:1591–6. 10.1016/j.dsx.2019.03.009 31336526

[41] Koda M Kawakami M Murawaki Y Senda M. The impact of visceral fat in nonalcoholic fatty liver disease: cross-sectional and longitudinal studies. *J Gastroenterol.* (2007) 42:897–903. 10.1007/s00535-007-2107-z 18008034

[42] Almahmoud MH Al Khawaja NM Alkinani A Khader Y Ajlouni KM. Prevalence of fatty liver disease and its associated factors among jordanian patients with type 2 diabetes mellitus: a cross-sectional study. *Ann Med Surg.* (2021) 68:102677. 10.1016/j.amsu.2021.102677 34401141 PMC8358152

[43] Properzi C O’Sullivan TA Sherriff JL Ching HL Jeffrey GP Buckley RF Ad libitum mediterranean and low-fat diets both significantly reduce hepatic steatosis: a randomized controlled trial. *Hepatology.* (2018) 68:1741–54. 10.1002/hep.30076 29729189

[44] Pang Q Zhang J-Y Song S-D Qu K Xu X-S Liu S-S Central obesity and nonalcoholic fatty liver disease risk after adjusting for body mass index. *World J Gastroenterol.* (2015) 21:1650–62. 10.3748/wjg.v21.i5.1650 25663786 PMC4316109

[45] Fan JG Saibara T Chitturi S Kim BI Sung JJ Chutaputti A. What are the risk factors and settings for non-alcoholic fatty liver disease in Asia-pacific? *J Gastroenterol Hepatol.* (2007) 22:794–800. 10.1111/j.1440-1746.2007.04952.x 17498218

[46] Buechler C Wanninger J Neumeier M. Adiponectin, a key adipokine in obesity related liver diseases. *World J Gastroenterol.* (2011) 17:2801–11. 10.3748/wjg.v17.i23.2801 21734787 PMC3120939

[47] Schäffler A Schölmerich J Büchler C. Mechanisms of disease: adipocytokines and visceral adipose tissue–emerging role in nonalcoholic fatty liver disease. *Nat Clin Pract Gastroenterol Hepatol.* (2005) 2:273–80. 10.1038/ncpgasthep0186 16265231

[48] Borst SE. The role of tnf-alpha in insulin resistance. *Endocrine.* (2004) 23:177–82. 10.1385/endo:23:2-3:177 15146098

[49] Kamada Y Takehara T Hayashi N. Adipocytokines and liver disease. *J Gastroenterol.* (2008) 43:811–22. 10.1007/s00535-008-2213-6 19012034

[50] Oguntibeju OO. Type 2 diabetes mellitus, oxidative stress and inflammation: examining the links. *Int J Physiol Pathophysiol Pharmacol.* (2019) 11:45–63. Epub 2019/07/25. 31333808 PMC6628012

[51] Shams MEE Al-Gayyar MMH Barakat EAME. Type 2 diabetes mellitus-induced hyperglycemia in patients with Nafld and normal Lfts: relationship to lipid profile, oxidative stress and pro-inflammatory cytokines. *Sci Pharm.* (2011) 79:623–34. 10.3797/scipharm.1104-21 21886908 PMC3163367

[52] Mansour-Ghanaei R Mansour-Ghanaei F Naghipour M Joukar F. Biochemical markers and lipid profile in nonalcoholic fatty liver disease patients in the persian guilan cohort study (Pgcs), Iran. *J Fam Med Prim Care.* (2019) 8:923–8. 10.4103/jfmpc.jfmpc_243_18 PMC6482810 31041226

[53] Jin HB Gu ZY Yu CH Li YM. Association of nonalcoholic fatty liver disease with type 2 diabetes: clinical features and independent risk factors in diabetic fatty liver patients. *Hepatobiliary Pancreat Dis Int.* (2005) 4:389–92. 16109522

[54] Mandal A Bhattarai B Kafle P Khalid M Jonnadula SK Lamicchane J Elevated liver enzymes in patients with type 2 diabetes mellitus and non-alcoholic fatty liver disease. *Cureus.* (2018) 10:e3626–e. 10.7759/cureus.3626 30697502 PMC6347442

[55] Armstrong MJ Houlihan DD Bentham L Shaw JC Cramb R Olliff S Presence and severity of non-alcoholic fatty liver disease in a large prospective primary care cohort. *J Hepatol.* (2012) 56:234–40. 10.1016/j.jhep.2011.03.020 21703178

[56] Després J-P Lemieux I. Abdominal obesity and metabolic syndrome. *Nature.* (2006) 444:881–7. 10.1038/nature05488 17167477

[57] Paschos P Paletas K. Non alcoholic fatty liver disease and metabolic syndrome. *Hippokratia.* (2009) 13:9–19. 19240815 PMC2633261

[58] Farrell GC Larter CZ. Nonalcoholic fatty liver disease: from steatosis to cirrhosis. *Hepatology.* (2006) 43:S99–112. 10.1002/hep.20973 16447287

[59] Gan L Chitturi S Farrell GC. Mechanisms and implications of age-related changes in the liver: nonalcoholic fatty liver disease in the elderly. *Curr Gerontol Geriatr Res.* (2011) 2011:831536. 10.1155/2011/831536 21918648 PMC3171768

[60] Guenther PM Kott PS Carriquiry AL. Development of an approach for estimating usual nutrient intake distributions at the population level. *J Nutr.* (1997) 127:1106–12. 10.1093/jn/127.6.1106 9187624

