# Supplementary material for: Higher carbohydrate intake in relation to non-alcoholic fatty liver disease in patients with type 2 diabetes
Source: Front Nutr. 2022 Dec 8;9:996004. doi: 10.3389/fnut.2022.996004 (PMC9773196; doi:10.3389/fnut.2022.996004)
Supplement: Supplementary file 1 [file Table_1.docx]

Supplementary Material

# Supplementary Figures and Tables

**Supplementary Table 1.** Stepwise multiple linear regression associations between carbohydrate intake as an independent variable and CAP values as a dependent variable in the total sample; participants ≤50 years of age and participants >50 years of age.

| **Model** | **Unstandardized coefficient** | | **Standardized coefficient** | **t** | ***P*-value** | **95% CI** | |
| --- | --- | --- | --- | --- | --- | --- | --- |
|  | **B** | Standard error |  |  |  | **Lower bound** | **Upper bound** |
| **In the total sample** | | | | | | | |
| **Model 1** (n = 358)  *P* = 0.367, R square = 0.009 | | | | | | | |
| Carbohydrate intake (%energy/day) | 0.512 | 0.393 | 0.071 | 1.303 | 0.193 | -0.261 | 1.285 |
| Age (years) | -0.522 | 0.421 | -0.066 | -1.239 | 0.216 | -1.349 | 0.306 |
| Sex | 5.008 | 6.341 | 0.043 | 0.790 | 0.430 | -7.462 | 17.479 |
| **Model 2** (n = 338)  *P* = 0.447, R square = 0.153 | | | | | | | |
| Carbohydrate intake (%energy/day) | 0.426 | 0.402 | 0.060 | 1.060 | 0.290 | -0.365 | 1.218 |
| Age (years) | 4.648 | 7.480 | 0.040 | 0.621 | 0.535 | -10.067 | 19.363 |
| Sex | -0.494 | 0.471 | -0.063 | -1.047 | 0.296 | -1.421 | 0.434 |
| Marital status | -5.825 | 4.337 | -0.076 | -1.343 | 0.180 | -14.356 | 2.706 |
| Level of Education | 3.457 | 1.886 | 0.110 | 1.833 | 0.068 | -0.253 | 7.168 |
| Employment | -0.302 | 2.025 | -0.009 | -0.149 | 0.882 | -4.286 | 3.683 |
| Average Family Income | 0.081 | 2.207 | 0.002 | 0.037 | 0.971 | -4.260 | 4.423 |
| Smoking | 6.939 | 11.574 | 0.035 | 0.600 | 0.549 | -15.829 | 29.707 |
| **Model 3** (n = 338)  *P* < 0.001, R square = 0.082 | | | | | | | |
| Carbohydrate intake (%energy/day) | 0.507 | 0.391 | 0.071 | 1.296 | 0.196 | -0.263 | 1.276 |
| Age (years) | 15.009 | 7.604 | 0.128 | 1.974 | 0.049 | 0.051 | 29.967 |
| Sex | -0.350 | 0.459 | -0.044 | -0.762 | 0.447 | -1.252 | 0.553 |
| Marital status | -6.189 | 4.211 | -0.081 | -1.470 | 0.143 | -14.472 | 2.094 |
| Level of Education | 3.323 | 1.831 | 0.106 | 1.814 | 0.071 | -0.280 | 6.925 |
| Employment | -1.249 | 1.977 | -0.038 | -0.632 | 0.528 | -5.138 | 2.640 |
| Average Family Income | 0.765 | 2.148 | 0.021 | 0.356 | 0.722 | -3.460 | 4.990 |
| Smoking | 5.340 | 11.241 | 0.027 | 0.475 | 0.635 | -16.773 | 27.453 |
| BMI (kg/m^2^) | 2.844 | 0.619 | 0.265 | 4.596 | <0.001 | 1.626 | 4.061 |
| **Model 4** ( n = 335)  *P* < 0.001, R square = 0.083 | | | | | | | |
| Carbohydrate intake (%energy/day) | 0.496 | 0.402 | 0.070 | 1.236 | 0.218 | -0.294 | 1.287 |
| Age (years) | 0.518 | 7.848 | 0.004 | 0.066 | 0.947 | -14.920 | 15.957 |
| Sex | -0.403 | 0.472 | -0.051 | -0.853 | 0.394 | -1.331 | 0.526 |
| Marital status | -5.598 | 4.268 | -0.074 | -1.311 | 0.191 | -13.994 | 2.799 |
| Level of Education | 2.890 | 1.874 | 0.092 | 1.542 | 0.124 | -0.798 | 6.577 |
| Employment | 0.314 | 2.009 | 0.010 | 0.157 | 0.876 | -3.638 | 4.267 |
| Average Family Income | -0.072 | 2.178 | -0.002 | -0.033 | 0.974 | -4.357 | 4.213 |
| Smoking | 2.504 | 11.469 | 0.013 | 0.218 | 0.827 | -20.060 | 25.067 |
| LDL-C (mmol/L) | -34.940 | 12.336 | -0.169 | -2.832 | 0.005 | -59.209 | -10.670 |
| HDL-C (mmol/L) | 8.243 | 3.539 | 0.131 | 2.329 | 0.020 | 1.281 | 15.205 |
| **Model 5** ( n = 334)  *P <* 0.001, R square = 0.083 | | | | | | | |
| Carbohydrate intake (%energy/day) | 0.429 | 0.393 | 0.060 | 1.091 | 0.276 | -0.344 | 1.202 |
| Age (years) | 5.588 | 7.304 | 0.048 | 0.765 | 0.445 | -8.782 | 19.958 |
| Sex | -0.467 | 0.461 | -0.059 | -1.014 | 0.311 | -1.374 | 0.439 |
| Marital status | -5.999 | 4.296 | -0.077 | -1.396 | 0.164 | -14.451 | 2.452 |
| Level of Education | 3.277 | 1.835 | 0.105 | 1.786 | 0.075 | -0.333 | 6.887 |
| Employment | 0.150 | 1.972 | 0.005 | 0.076 | 0.939 | -3.729 | 4.030 |
| Average Family Income | 0.075 | 2.155 | 0.002 | 0.035 | 0.972 | -4.164 | 4.314 |
| Smoking | 1.665 | 11.284 | 0.008 | 0.148 | 0.883 | -20.533 | 23.863 |
| Triglycerides (mmol/L) | 17.939 | 3.907 | 0.246 | 4.592 | <0.001 | 10.253 | 25.625 |
| **In participants aged ≤50 years** | | | | | | | |
| **Model 1** (n = 163)  *P* = 0.104, R square = 0.028 | | | | | | | |
| Carbohydrate intake (%energy/day) | 0.995 | 0.593 | 0.134 | 1.679 | 0.095 | -0.175 | 2.165 |
| Sex | 15.541 | 9.378 | 0.132 | 1.657 | 0.099 | -2.979 | 34.062 |
| **Model 2** (n = 154)  *P* = 0.93, R square = 0.079 | | | | | | | |
| Carbohydrate intake (%energy/day) | 1.124 | 0.609 | 0.153 | 1.846 | 0.067 | -0.080 | 2.327 |
| Sex | 6.805 | 11.581 | 0.058 | 0.588 | 0.558 | -16.084 | 29.693 |
| Marital status | -11.481 | 6.398 | -0.146 | -1.794 | 0.075 | -24.126 | 1.164 |
| Level of Education | 4.879 | 2.674 | 0.153 | 1.825 | 0.070 | -0.405 | 10.164 |
| Employment | -5.314 | 3.205 | -0.152 | -1.658 | 0.100 | -11.649 | 1.021 |
| Average Family Income | -0.290 | 3.095 | -0.008 | -0.094 | 0.925 | -6.406 | 5.826 |
| Smoking | 12.489 | 17.989 | 0.059 | 0.694 | 0.489 | -23.064 | 48.042 |
| **Model 3** (n = 154)  *P* < 0.001, R square = 0.165 | | | | | | | |
| Carbohydrate intake (%energy/day) | 1.249 | 0.583 | 0.171 | 2.143 | 0.034 | 0.097 | 2.401 |
| Sex | 18.100 | 11.450 | 0.154 | 1.581 | 0.116 | -4.531 | 40.731 |
| Marital status | -10.804 | 6.118 | -0.137 | -1.766 | 0.079 | -22.895 | 1.287 |
| Level of Education | 3.830 | 2.570 | 0.120 | 1.490 | 0.138 | -1.249 | 8.909 |
| Employment | -7.341 | 3.108 | -0.211 | -2.362 | 0.020 | -13.484 | -1.197 |
| Average Family Income | 0.378 | 2.963 | 0.010 | 0.127 | 0.899 | -5.478 | 6.233 |
| Smoking | 8.802 | 17.220 | 0.042 | 0.511 | 0.610 | -25.232 | 42.835 |
| BMI (kg/m^2^) | 3.243 | 0.842 | 0.326 | 3.852 | <0.001 | 1.579 | 4.907 |
| **Model 4** (n = 153)  *P* = 0.008, R square = 0.141 | | | | | | | |
| Carbohydrate intake (%energy/day) | 1.400 | 0.618 | 0.191 | 2.267 | 0.025 | 0.180 | 2.621 |
| Sex | 1.750 | 12.533 | 0.015 | 0.140 | 0.889 | -23.024 | 26.525 |
| Marital status | -10.843 | 6.267 | -0.137 | -1.730 | 0.086 | -23.232 | 1.545 |
| Level of Education | 4.157 | 2.618 | 0.131 | 1.588 | 0.115 | -1.018 | 9.332 |
| Employment | -4.748 | 3.147 | -0.136 | -1.509 | 0.134 | -10.969 | 1.473 |
| Average Family Income | -0.172 | 3.022 | -0.005 | -0.057 | 0.955 | -6.145 | 5.800 |
| Smoking | 4.932 | 17.707 | 0.023 | 0.279 | 0.781 | -30.069 | 39.934 |
| LDL-C (mmol/L) | 11.074 | 4.795 | 0.187 | 2.309 | 0.022 | 1.595 | 20.553 |
| HDL-C (mmol/L) | -38.685 | 18.448 | -0.185 | -2.097 | 0.038 | -75.152 | -2.219 |
| **Model 5** (n = 152)  *P* = 0.007, R square = 0.135 | | | | | | | |
| Carbohydrate intake (%energy/day) | 1.170 | 0.600 | 0.159 | 1.949 | 0.053 | -0.017 | 2.357 |
| Sex | 8.403 | 11.443 | 0.071 | 0.734 | 0.464 | -14.216 | 31.022 |
| Marital status | -11.586 | 6.611 | -0.140 | -1.753 | 0.082 | -24.654 | 1.482 |
| Level of Education | 5.061 | 2.625 | 0.159 | 1.928 | 0.056 | -0.127 | 10.249 |
| Employment | -4.889 | 3.155 | -0.140 | -1.549 | 0.123 | -11.126 | 1.348 |
| Average Family Income | -0.849 | 3.056 | -0.023 | -0.278 | 0.781 | -6.890 | 5.191 |
| Smoking | 6.705 | 17.710 | 0.032 | 0.379 | 0.706 | -28.302 | 41.713 |
| Triglycerides (mmol/L) | 15.788 | 5.443 | 0.228 | 2.901 | 0.004 | 5.029 | 26.546 |
| **In participants aged >50 years** | | | | | | | |
| **Model 1** (n = 195)  *P* = 0.89, R square = 0.001 | | | | | | | |
| Carbohydrate intake (%energy/day) | 0.066 | 0.522 | 0.009 | 0.127 | 0.899 | -0.964 | 1.096 |
| Sex | -3.780 | 8.668 | -.032 | -.436 | 0.663 | -20.878 | 13.317 |
| **Model 2** (n = 184)  *P* = 0.982, R square = 0.008 | | | | | | | |
| Carbohydrate intake (%energy/day) | 0.001 | 0.551 | 0.000 | 0.001 | 0.999 | -1.087 | 1.088 |
| Sex | -0.487 | 10.548 | -0.004 | -0.046 | 0.963 | -21.303 | 20.329 |
| Marital status | -2.390 | 6.012 | -0.032 | -0.398 | 0.691 | -14.254 | 9.474 |
| Level of Education | 1.823 | 2.736 | 0.059 | 0.666 | 0.506 | -3.577 | 7.224 |
| Employment | 1.552 | 2.674 | 0.045 | 0.580 | 0.562 | -3.726 | 6.829 |
| Average Family Income | -0.529 | 3.297 | -0.015 | -0.160 | 0.873 | -7.036 | 5.979 |
| Smoking | 3.494 | 15.471 | 0.018 | 0.226 | 0.822 | -27.038 | 34.027 |
| **Model 3** (n = 184)  *P* = 0.265, R square = 0.055 | | | | | | | |
| Carbohydrate intake (%energy/day) | 0.109 | 0.541 | 0.015 | .201 | .841 | -.959 | 1.176 |
| Sex | 7.938 | 10.723 | 0.067 | 0.740 | 0.460 | -13.224 | 29.100 |
| Marital status | -3.496 | 5.899 | -0.047 | -0.593 | 0.554 | -15.138 | 8.146 |
| Level of Education | 2.481 | 2.689 | 0.080 | 0.923 | 0.357 | -2.826 | 7.788 |
| Employment | 1.397 | 2.619 | 0.041 | 0.533 | 0.595 | -3.772 | 6.565 |
| Average Family Income | 0.724 | 3.257 | 0.020 | 0.222 | 0.824 | -5.704 | 7.152 |
| Smoking | 4.147 | 15.151 | 0.021 | 0.274 | 0.785 | -25.755 | 34.049 |
| BMI (kg/m^2^) | 2.701 | 0.924 | 0.233 | 2.925 | 0.004 | 0.878 | 4.524 |
| **Model 4** (n = 182)  *P* = 0.832, R square = 0.028 | | | | | | | |
| Carbohydrate intake (%energy/day) | -0.023 | 0.548 | -0.003 | -0.041 | 0.967 | -1.105 | 1.060 |
| Sex | -3.300 | 10.802 | -0.028 | -0.306 | 0.760 | -24.622 | 18.021 |
| Marital status | -2.006 | 5.969 | -0.027 | -0.336 | 0.737 | -13.788 | 9.776 |
| Level of Education | 1.070 | 2.785 | 0.034 | 0.384 | 0.701 | -4.426 | 6.567 |
| Employment | 2.296 | 2.700 | 0.067 | 0.850 | 0.396 | -3.033 | 7.625 |
| Average Family Income | -1.143 | 3.308 | -0.033 | -0.346 | 0.730 | -7.673 | 5.386 |
| Smoking | -0.346 | 15.463 | -0.002 | -0.022 | 0.982 | -30.867 | 30.175 |
| LDL-C (mmol/L) | 4.934 | 5.437 | 0.072 | 0.908 | 0.365 | -5.797 | 15.665 |
| HDL-C (mmol/L) | -31.182 | 17.097 | -0.152 | -1.824 | 0.070 | -64.930 | 2.565 |
| **Model 5** (n = 182)  *P* = 0.104, R square = 0.072 | | | | | | | |
| Carbohydrate intake (%energy/day) | -0.021 | 0.535 | -0.003 | -0.040 | 0.968 | -1.077 | 1.035 |
| Sex | -0.077 | 10.222 | -0.001 | -0.008 | 0.994 | -20.252 | 20.097 |
| Marital status | -3.321 | 5.825 | -0.045 | -0.570 | 0.569 | -14.818 | 8.175 |
| Level of Education | 1.479 | 2.651 | 0.048 | 0.558 | 0.578 | -3.755 | 6.712 |
| Employment | 1.972 | 2.589 | 0.058 | 0.762 | 0.447 | -3.138 | 7.081 |
| Average Family Income | -0.221 | 3.194 | -0.006 | -0.069 | 0.945 | -6.526 | 6.083 |
| Smoking | -1.754 | 14.993 | -0.009 | -0.117 | 0.907 | -31.346 | 27.839 |
| Triglycerides (mmol/L) | 19.651 | 5.661 | 0.256 | 3.471 | <0.001 | 8.477 | 30.825 |

*Note: p < 0.05 is considered significant. CAP, controlled attenuation parameter; CI, confidence interval; BMI, body mass index; HDL-C, high-density lipoprotein cholesterol; LDL-C, low-density lipoprotein cholesterol.*

**Supplementary Table 2.** Logistic regression models of carbohydrate intake as an independent variable and NAFLD presence as a dependent variable in the total study sample.

| **Model** | **B** | **S.E.** | **OR** | | ***P*-value** | **95% CI** | |
| --- | --- | --- | --- | --- | --- | --- | --- |
|  |  |  |  |  |  | **Lower bound** | **Upper bound** |
| **Model 1** (n = 358)  X^2^ (3, *N* = 338) = 6.639, *P* = 0.084 | | | | | | | |
| Carbohydrate intake (%energy/day) | 0.016 | 0.017 | 1.016 | | 0.350 | 0.983 | 1.049 |
| Age (years) | -0.021 | 0.018 | 0.979 | | 0.243 | 0.945 | 1.015 |
| Sex | -0.517 | 0.273 | 0.596 | | 0.058 | 0.349 | 1.017 |
| **Model 2** (n = 338)  X^2^ (8, *N* = 338) = 5.495, *P* = 0.704 | | | | | | | |
| Carbohydrate intake (%energy/day) | 0.013 | 0.018 | 1.013 | | 0.479 | 0.977 | 1.050 |
| Age (years) | -0.030 | 0.024 | 0.971 | | 0.212 | 0.927 | 1.017 |
| Sex | -1.057 | 0.502 | 0.348 | | 0.035 | 0.130 | 0.929 |
| Marital status |  |  |  |  |  |  |  |
| Married (ref.) |  |  |  |  | 0.033 |  |  |
| Single | 0.365 | 1.181 | 1.441 | | 0.757 | 0.142 | 14.594 |
| Divorced | 1.350 | 1.089 | 3.858 | | 0.215 | 0.456 | 32.642 |
| Widowed | -1.498 | 0.591 | 0.224 | | 0.011 | 0.070 | 0.713 |
| Level of Education |  |  |  |  |  |  |  |
| High school (ref.) |  |  |  |  | 0.530 |  |  |
| College/University | -0.690 | 0.433 | 0.501 | | 0.111 | 0.214 | 1.172 |
| Master's degree | -0.456 | 0.852 | 0.634 | | 0.592 | 0.119 | 3.369 |
| Doctorate | 19.933 | 17771.716 | 453887598 | | 0.999 | 0 | . |
| Less than high school | -0.721 | 0.491 | 0.486 | | 0.142 | 0.186 | 1.273 |
| Not specified | -0.102 | 0.586 | 0.903 | | 0.861 | 0.286 | 2.848 |
| Employment |  |  |  |  |  |  |  |
| Student (ref.) |  |  |  |  | 0.863 |  |  |
| Public sector | 0.818 | 1.012 | 2.266 | | 0.419 | 0.312 | 16.459 |
| Private sector | 0.486 | 1.201 | 1.626 | | 0.685 | 0.155 | 17.115 |
| Freelancer | 0.659 | 1.204 | 1.932 | | 0.584 | 0.183 | 20.442 |
| Housewife | 0.351 | 1.090 | 1.420 | | 0.748 | 0.168 | 12.033 |
| Does not work | 0.959 | 1.092 | 2.610 | | 0.380 | 0.307 | 22.184 |
| Others/Retired | 1.283 | 1.160 | 3.609 | | 0.268 | 0.372 | 35.039 |
| Average Family Income |  |  |  |  |  |  |  |
| <5000 SR (ref.) |  |  |  |  | 0.957 |  |  |
| 5000-10000 SR | 0.196 | 0.558 | 1.217 | | 0.725 | 0.407 | 3.635 |
| 10000-15000 SR | -0.439 | 0.563 | 0.645 | | 0.435 | 0.214 | 1.943 |
| 15000-20000 SR | -0.112 | 0.610 | 0.894 | | 0.855 | 0.270 | 2.958 |
| 20000-30000 SR | -0.147 | 0.641 | 0.863 | | 0.818 | 0.246 | 3.034 |
| 30000-40000 SR | 0.435 | 0.962 | 1.545 | | 0.651 | 0.234 | 10.178 |
| >40000 SR | -0.360 | 0.892 | 0.698 | | 0.686 | 0.122 | 4.005 |
| Do not know | -23.548 | 40193 | 0 | | 1.000 | 0 | . |
| Refused to answer | 19.087 | 40193 | 194653443 | | 1.000 | 0 | . |
| Smoking | 0.512 | 0.520 | 1.668 | | 0.325 | 0.602 | 4.624 |
| **Model 3** (n = 338)  X^2^ (27, *N* = 338) = 41.784, *P* = 0.035 | | | | | | | |
| Carbohydrate intake (%energy/day) | 0.016 | 0.019 | 1.016 | | 0.386 | 0.980 | 1.054 |
| Age (years) | -0.028 | 0.024 | 0.973 | | 0.258 | 0.927 | 1.020 |
| Sex | -0.842 | 0.516 | 0.431 | | 0.103 | 0.157 | 1.184 |
| Marital status |  |  |  | |  |  |  |
| Married (ref.) |  |  |  | | 0.021 |  |  |
| Single | 0.615 | 1.251 | 1.849 | | 0.623 | 0.159 | 21.449 |
| Divorced | 1.367 | 1.118 | 3.923 | | 0.222 | 0.438 | 35.098 |
| Widowed | -1.638 | 0.604 | 0.194 | | 0.007 | 0.060 | 0.635 |
| Level of Education |  |  |  | |  |  |  |
| High school (ref.) |  |  |  | | 0.731 |  |  |
| College/University | -0.520 | 0.442 | 0.595 | | 0.239 | 0.250 | 1.413 |
| Master's degree | -0.385 | 0.858 | 0.681 | | 0.654 | 0.127 | 3.658 |
| Doctorate | 19.838 | 17732 | 412414795 | | 0.999 | 0 | . |
| Less than high school | -0.667 | 0.502 | 0.513 | | 0.184 | 0.192 | 1.373 |
| Not specified | -0.083 | 0.596 | 0.920 | | 0.889 | 0.286 | 2.961 |
| Employment |  |  |  | |  |  |  |
| Student (ref.) |  |  |  | | 0.826 |  |  |
| Public sector | 0.809 | 0.999 | 2.246 | | 0.418 | 0.317 | 15.919 |
| Private sector | 0.518 | 1.205 | 1.679 | | 0.667 | 0.158 | 17.811 |
| Freelancer | 0.642 | 1.201 | 1.899 | | 0.593 | 0.180 | 19.992 |
| Housewife | 0.135 | 1.083 | 1.145 | | 0.901 | 0.137 | 9.564 |
| Does not work | 0.882 | 1.085 | 2.416 | | 0.416 | 0.288 | 20.273 |
| Others/Retired | 1.213 | 1.155 | 3.362 | | 0.294 | 0.350 | 32.332 |
| Average Family Income |  |  |  | |  |  |  |
| <5000 SR (ref.) |  |  |  | | 0.950 |  |  |
| 5000-10000 SR | 0.157 | 0.570 | 1.170 | | 0.784 | 0.382 | 3.577 |
| 10000-15000 SR | -0.529 | 0.578 | 0.589 | | 0.360 | 0.190 | 1.829 |
| 15000-20000 SR | -0.197 | 0.619 | 0.821 | | 0.750 | 0.244 | 2.760 |
| 20000-30000 SR | -0.185 | 0.648 | 0.831 | | 0.776 | 0.233 | 2.962 |
| 30000-40000 SR | 0.352 | 0.978 | 1.422 | | 0.719 | 0.209 | 9.673 |
| >40000 SR | -0.340 | 0.906 | 0.712 | | 0.708 | 0.121 | 4.204 |
| Do not know | -23.831 | 40193 | 0 | | 1.000 | 0 | . |
| Refused to answer | 19.298 | 40193 | 240514833 | | 1.000 | 0 | . |
| Smoking | 0.477 | 0.530 | 1.611 | | 0.368 | 0.570 | 4.553 |
| BMI | 0.099 | 0.034 | 1.104 | | 0.003 | 1.033 | 1.180 |
| **Model 4** ( n = 335)  X^2^ (28, *N* = 335) = 41.943 , *P* = 0.044 | | | | | | | |
| Carbohydrate intake (%energy/day) | 0.008 | 0.019 | 1.008 | | 0.657 | 0.971 | 1.047 |
| Age (years) | -0.031 | 0.024 | 0.969 | | 0.203 | 0.924 | 1.017 |
| Sex | -1.323 | 0.536 | 0.266 | | 0.014 | 0.093 | 0.763 |
| Marital status |  |  |  | |  |  |  |
| Married (ref.) |  |  |  | | 0.047 |  |  |
| Single | 0.134 | 1.189 | 1.143 | | 0.910 | 0.111 | 11.751 |
| Divorced | 1.369 | 1.105 | 3.931 | | 0.215 | 0.451 | 34.293 |
| Widowed | -1.454 | 0.603 | 0.234 | | 0.016 | 0.072 | 0.762 |
| Level of Education |  |  |  | |  |  |  |
| High school (ref.) |  |  |  | | 0.342 |  |  |
| College/University | -0.852 | 0.454 | 0.427 | | 0.061 | 0.175 | 1.039 |
| Master's degree | -0.825 | 0.869 | 0.438 | | 0.343 | 0.080 | 2.408 |
| Doctorate | 20 | 17876 | 486798386 | | 0.999 | 0 | . |
| Less than high school | -0.939 | 0.511 | 0.391 | | 0.066 | 0.144 | 1.065 |
| Not specified | -0.185 | 0.609 | 0.831 | | 0.761 | 0.252 | 2.742 |
| Employment |  |  |  | |  |  |  |
| Student (ref.) |  |  |  | | 0.831 |  |  |
| Public sector | 0.765 | 1.037 | 2.150 | | 0.461 | 0.282 | 16.414 |
| Private sector | 0.365 | 1.228 | 1.440 | | 0.767 | 0.130 | 15.989 |
| Freelancer | 0.605 | 1.237 | 1.832 | | 0.625 | 0.162 | 20.714 |
| Housewife | 0.470 | 1.116 | 1.601 | | 0.673 | 0.180 | 14.275 |
| Does not work | 1.082 | 1.133 | 2.951 | | 0.339 | 0.320 | 27.185 |
| Others/Retired | 1.356 | 1.186 | 3.880 | | 0.253 | 0.379 | 39.681 |
| Average Family Income |  |  |  | |  |  |  |
| <5000 SR (ref.) |  |  |  | | 0.969 |  |  |
| 5000-10000 SR | 0.160 | 0.576 | 1.174 | | 0.781 | 0.380 | 3.626 |
| 10000-15000 SR | -0.456 | 0.582 | 0.634 | | 0.433 | 0.203 | 1.981 |
| 15000-20000 SR | -0.092 | 0.647 | 0.912 | | 0.887 | 0.257 | 3.241 |
| 20000-30000 SR | -0.230 | 0.666 | 0.794 | | 0.730 | 0.215 | 2.929 |
| 30000-40000 SR | 0.286 | 1.007 | 1.331 | | 0.777 | 0.185 | 9.578 |
| >40000 SR | -0.453 | 0.913 | 0.636 | | 0.619 | 0.106 | 3.802 |
| Do not know | -23.152 | 40193 | 0 | | 1.000 | 0 | . |
| Refused to answer | 18.720 | 40193 | 134944538 | | 1.000 | 0 | . |
| Smoking | 0.337 | 0.533 | 1.401 | | 0.527 | 0.492 | 3.984 |
| LDL-C | 0.206 | 0.179 | 1.228 | | 0.250 | 0.865 | 1.744 |
| HDL-C | -1.712 | 0.585 | 0.180 | | 0.003 | 0.057 | 0.568 |
| **Model 5** ( n = 334)  X^2^ (27, *N* = 334) = 44.191 , *P* = 0.020 | | | | | | | |
| Carbohydrate intake (%energy/day) | 0.012 | 0.019 | 1.012 | | 0.525 | 0.975 | 1.050 |
| Age (years) | -0.027 | 0.024 | 0.974 | | 0.269 | 0.928 | 1.021 |
| Sex | -0.987 | 0.519 | 0.373 | | 0.057 | 0.135 | 1.030 |
| Marital status |  |  |  | |  |  |  |
| Married (ref.) |  |  |  | | 0.015 |  |  |
| Single | 0.362 | 1.181 | 1.436 | | 0.759 | 0.142 | 14.552 |
| Divorced | 1.461 | 1.097 | 4.312 | | 0.183 | 0.502 | 37.048 |
| Widowed | -1.727 | 0.620 | 0.178 | | 0.005 | 0.053 | 0.600 |
| Level of Education |  |  |  | |  |  |  |
| High school (ref.) |  |  |  | | 0.414 |  |  |
| College/University | -0.819 | 0.456 | 0.441 | | 0.072 | 0.180 | 1.078 |
| Master's degree | -0.796 | 0.854 | 0.451 | | 0.351 | 0.085 | 2.406 |
| Doctorate | 19.362 | 17631 | 256399007 | | 0.999 | 0 | . |
| Less than high school | -0.760 | 0.509 | 0.468 | | 0.135 | 0.173 | 1.268 |
| Not specified | -0.084 | 0.603 | 0.920 | | 0.890 | 0.282 | 2.998 |
| Employment |  |  |  | |  |  |  |
| Student (ref.) |  |  |  | | 0.817 |  |  |
| Public sector | 0.669 | 1.051 | 1.952 | | 0.524 | 0.249 | 15.308 |
| Private sector | 0.155 | 1.242 | 1.168 | | 0.901 | 0.102 | 13.316 |
| Freelancer | 0.416 | 1.253 | 1.516 | | 0.740 | 0.130 | 17.659 |
| Housewife | 0.267 | 1.127 | 1.305 | | 0.813 | 0.143 | 11.884 |
| Does not work | 0.960 | 1.135 | 2.611 | | 0.398 | 0.282 | 24.149 |
| Others/Retired | 1.164 | 1.198 | 3.201 | | 0.332 | 0.306 | 33.518 |
| Average Family Income |  |  |  | |  |  |  |
| <5000 SR (ref.) |  |  |  | | 0.988 |  |  |
| 5000-10000 SR | 0.367 | 0.577 | 1.443 | | 0.524 | 0.466 | 4.468 |
| 10000-15000 SR | -0.175 | 0.584 | 0.839 | | 0.764 | 0.267 | 2.637 |
| 15000-20000 SR | 0.174 | 0.641 | 1.191 | | 0.786 | 0.339 | 4.186 |
| 20000-30000 SR | -0.006 | 0.668 | 0.994 | | 0.992 | 0.268 | 3.680 |
| 30000-40000 SR | 0.397 | 1.002 | 1.487 | | 0.692 | 0.209 | 10.593 |
| >40000 SR | -0.090 | 0.905 | 0.914 | | 0.921 | 0.155 | 5.389 |
| Do not know | -22.989 | 40193 | 0 | | 1.000 | 0 | . |
| Refused to answer | 19.194 | 40193 | 216701215 | | 1.000 | 0 | . |
| Smoking | 0.369 | 0.539 | 1.447 | | 0.493 | 0.503 | 4.160 |
| Triglycerides | 0.732 | 0.255 | 2.079 | | 0.004 | 1.261 | 3.426 |

*Note: p < 0.05 is considered significant. Ref., reference category; OR, odds ratio; CI, confidence interval; BMI, body mass index; HDL-C, high-density lipoprotein cholesterol; LDL-C, low-density lipoprotein cholesterol; SR, Saudi riyals. The reference category for the variable “sex” is “female”. The reference category for the variable “smoking” is “non-smoker”.*

**Supplementary Table 3.** Receiver operating characteristic (ROC) curve analysis results of the total study sample, participants aged ≤50 years, and participants aged >50 years using carbohydrate intake (%energy/day) as the predictive variable and the presence of NAFLD as the outcome.

| **Model** | **Area under the curve** | **95% CI** | | ***P*-value** | **Cutoff point** | **Sensitivity** | | **Specificity** | |  |
| --- | --- | --- | --- | --- | --- | --- | --- | --- | --- | --- |
| **In the total sample ^1^** | | | | | | | | | |  |
| Unadjusted | 0.535 | 0.460 - 0.611 | 0.347 | | 60% | | 40.5% | | 63.5% | |
| Model 1 | 0.599 | 0.523 - 0.676 | 0.008 | | 70% | | 96.8% | | 5.4% | |
| Model 2 | 0.721 | 0.655 - 0.787 | < 0.001 | | 60.3% | | 97.4% | | 15.9% | |
| Model 3 | 0.747 | 0.682 - 0.813 | < 0.001 | | 60.3% | | 95.5% | | 29% | |
| Model 4 | 0.757 | 0.693 - 0.822 | < 0.001 | | 60.5% | | 94.8% | | 20.6% | |
| Model 5 | 0.750 | 0.688 - 0.812 | < 0.001 | | 60.4% | | 94.4% | | 23.5% | |
| **In participants aged ≤50 years ^2^** | | | | | | | | |  | |
| Unadjusted | 0.558 | 0.447 - 0.670 | 0.057 | | 55% | | 64.3% | | 41.2% | |
| Model 1 | 0.557 | 0.444 - 0.669 | 0.311 | | 63.7% | | 100% | | 2.9% | |
| Model 2 | 0.802 | 0.712 - 0.892 | < 0.001 | | 60% | | 93.4% | | 53.1% | |
| Model 3 | 0.820 | 0.734 - 0.906 | < 0.001 | | 60.5% | | 92.6% | | 53.1% | |
| Model 4 | 0.827 | 0.743 - 0.911 | < 0.001 | | 60.3% | | 92.6% | | 53.1% | |
| Model 5 | 0.821 | 0.732 - 0.910 | < 0.001 | | 60.1% | | 94.2% | | 62.5% | |
| **In participants aged >50 years ^2^** | | | | | | | | |  | |
| Unadjusted | 0.519 | 0.417 - 0.621 | 0.711 | | 55% | | 63.9% | | 40% | |
| Model 1 | 0.620 | 0.525 - 0.716 | 0.019 | | 71.2% | | 96.1% | | 2.5% | |
| Model 2 | 0.753 | 0.667 - 0.839 | < 0.001 | | 61.4% | | 95.9% | | 27% | |
| Model 3 | 0.787 | 0.710 - 0.864 | < 0.001 | | 61% | | 93.2% | | 32.4% | |
| Model 4 | 0.818 | 0.748 - 0.887 | < 0.001 | | 60% | | 91.1% | | 33.3% | |
| Model 5 | 0.794 | 0.716 - 0.872 | < 0.001 | | 60% | | 92.5% | | 33.3% | |

*Note: p < 0.05 is considered significant; CI, confidence interval.*

*^1^Model 1 is adjusted for age and sex, model 2 is additionally adjusted for demographic data and smoking, model 3 is additionally adjusted for body mass index (BMI), model 4 is model 2 adjusted for high-density lipoprotein cholesterol (HDL-C) and low-density lipoprotein cholesterol (LDL-C), model 5 is model 2 adjusted for triglycerides.*

*^2^Model 1 is adjusted for sex, model 2 is additionally adjusted for demographic data and smoking, model 3 is additionally adjusted for BMI, model 4 is model 2 adjusted for HDL-C and LDL-C, model 5 is model 2 adjusted for triglycerides.*


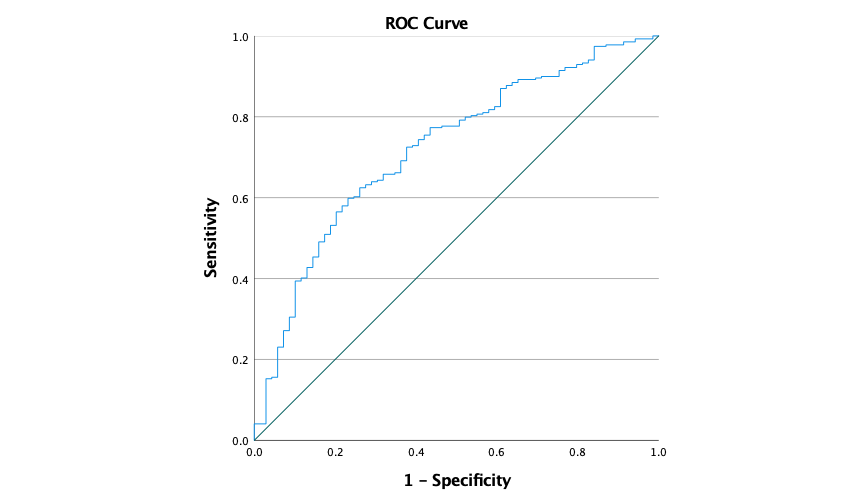


**Supplementary Figure 1.** Receiver operating characteristic (ROC) curve of model 2 for the total sample suggests acceptable discrimination between outcome groups (AUC = 0.721, 95%CI = 0.655-0.787, *P* = < 0.001).


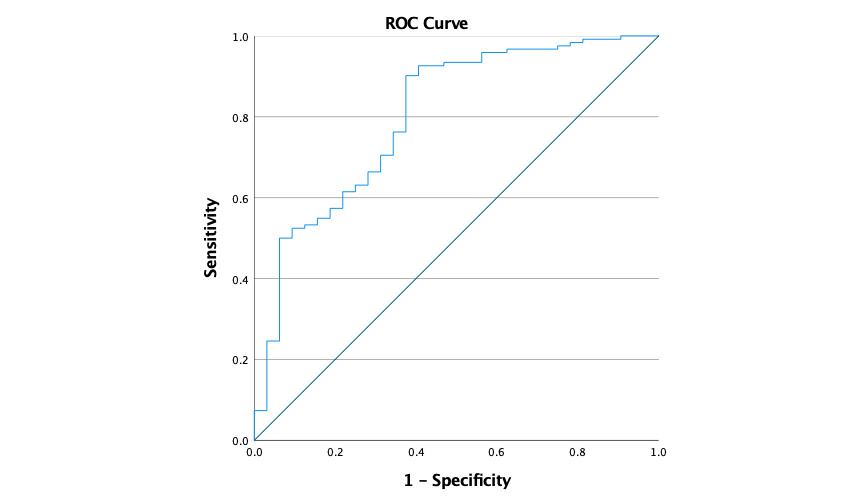


**Supplementary Figure 2.** Receiver operating characteristic (ROC) curve of model 2 for participants aged 50 years and below suggests acceptable discrimination between outcome groups (AUC = 0.802, 95%CI = 0.712-0.892, *P* = < 0.001).


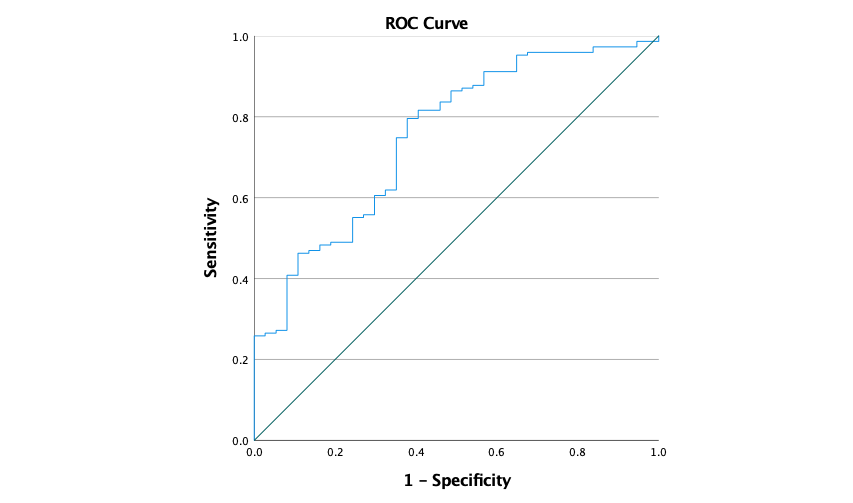


**Supplementary Figure 3.** Receiver operating characteristic (ROC) curve of model 2 for participants aged >50 years suggests acceptable discrimination between outcome groups (AUC = 0.753, 95%CI 0.667-0.839, *P* = < 0.001).
